# Supplementary material for: Antimicrobial Activity of Chamomile Essential Oil: Effect of Different Formulations
Source: Molecules. 2019 Nov 26;24(23):4321. doi: 10.3390/molecules24234321 (PMC6930572; doi:10.3390/molecules24234321)
Supplement: Supplementary file 1 [file molecules-24-04321-s001.pdf]

Antimicrobial activity of chamomile essential oil: Effect of different formulations

Supplementary Material

Table S1. Results of GC-MS analysis of chamomile EO. In the table the major components can be seen, the compounds that were present significantly under 1% was not indicated or identified.

No.	Compound	t_R (min)	Area percentage (%)
1	trans β-Farnesene	23.304	17.93
2	Germacrene D	23.971	1.54
3	bicyclogermacrene	24.526	1.39
4	(E,E)-α-Farnesene	25.100	2.08
5	spathulenol	27.676	1.88
6	Bisabolol oxide B	30.732	7.57
7	Bisabolone	32.133	21.83
8.	Chamazulen	33.375	3.19
9.	Bisabolol oxide A	34.127	10.48
10.	Vetivazulene	35.048	4.94

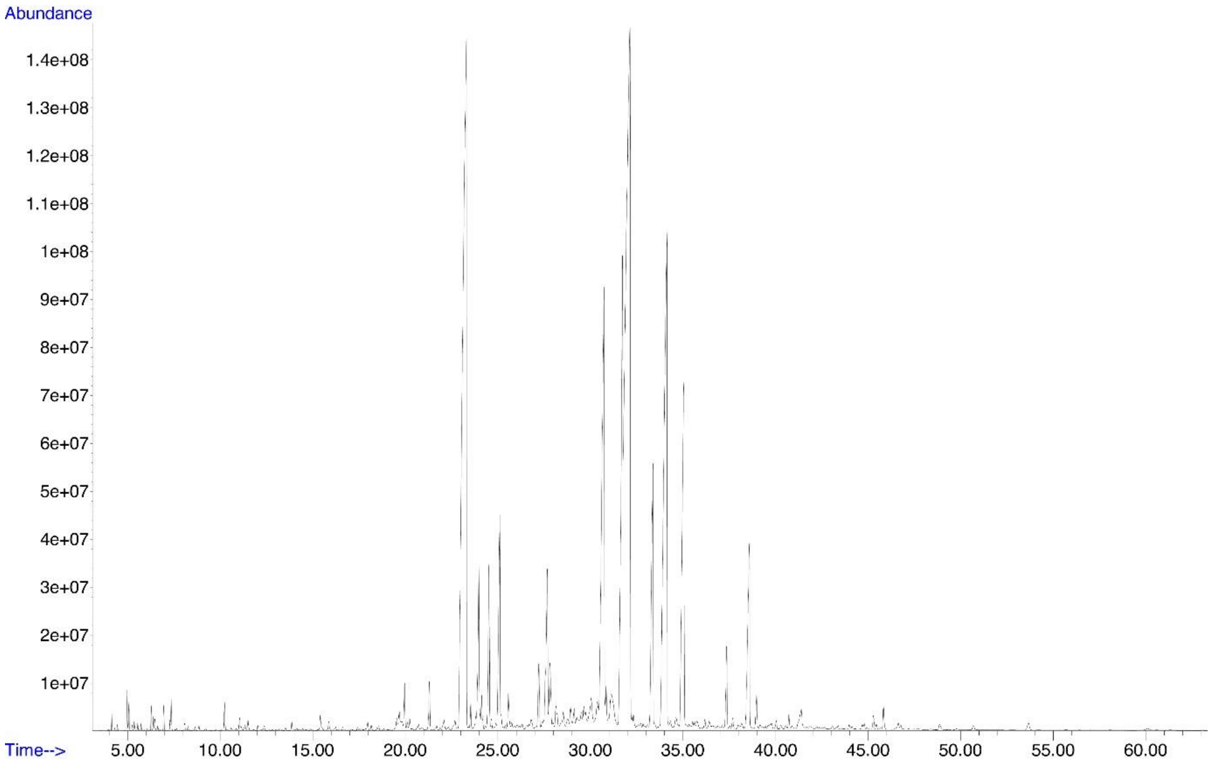

Figure S1. Chromatogram of chamomile essential oil obtained by GC/MS.
